# The mixed subtype has a worse prognosis than other histological subtypes: a retrospective analysis of 217 patients with metaplastic breast cancer

**DOI:** 10.1007/s10549-023-06945-9

**Published:** 2023-05-09

**Authors:** Jiayue Hu, Ronggang Lang, Weipeng Zhao, Yongsheng Jia, Zhongsheng Tong, Yehui Shi

**Affiliations:** 1 Department of Breast Oncology, National Clinical Research Center of Cancer, Key Laboratory of Cancer Prevention and Therapy, Key Laboratory of Breast Cancer Prevention and Therapy, Tianjin Medical University Cancer Institute and Hospital, Tianjin, 300060 China; 2 Department of Breast Pathology and Lab, Department of Breast Oncology, National Clinical Research Center of Cancer, Key Laboratory of Breast Cancer of Breast Cancer Prevention and Therapy, Tianjin Medical University Cancer Institute and Hospital, Tianjin, 300060 China

**Keywords:** Metaplastic breast cancer, Mixed histological subtype, Squamous, Spindle, Propensity score matching

## Abstract

**Objective:**

Metaplastic breast cancer (MpBC) is an aggressive subtype of all breast cancer. We aimed to investigate the clinicopathological features, treatments and prognoses of MpBC patients.

**Methods:**

We collected the data from MpBC patients diagnosed at Tianjin Medical University Cancer Hospital from 2010 to 2017. Kaplan Meier curves and Cox regression model were used to evaluating clinical outcomes and prognostic factors. After removing baseline differences by propensity score matching (PSM), we analyzed the prognosis between MpBC patients and invasive ductal carcinomas of no special type (IDC-NST) patients.

**Results:**

A total of 217 MpBC patients were subsumed. Of all histological subtypes, 45.1% were mixed subtypes, followed by with mesenchymal differentiation (27.2%), pure squamous (15.2%) and pure spindle (12.4%) subtypes. 69.6% of MpBC were triple-negative, 25.3% and 6.5% were HR-positive and HER2-positive. MpBC patients had worse survival compared to IDC-NST patients, with 5-year RFS of 73.8 and 83.6% (HR = 1.177 95%CI (1.171–2.676) *P* = 0.0068), and 5-year BCSS of 79.0% and 89.7% (HR = 2.187 95%CI (1.357–3.523) *P* = 0.0013). In the multivariate COX model, AJCC stage, mixed subtype and chemotherapy were independent prognostic factors. Mixed MpBC is more aggressive than pure and with heterologous mesenchymal differentiation subtypes. And whether squamous or spindle MpBC, mixed forms have shorter outcomes than pure forms.

**Conclusions:**

MpBCs are associated with poorer prognoses than IDC-NSTs. They are heterogeneous with different clinicopathological features and clinical outcomes between histological subtypes. Pure and with heterologous mesenchymal differentiation subtypes have more survival benefits than the mixed subtype.

## Introduction

MpBC is a rare subtype that constitutes 0.2–5% of all breast cancer [1]. It has heterogeneity which features the transformation of the neoplastic cells toward epithelial or (and) mesenchymal components. The 5th edition of the WHO classification includes six subtypes: pure squamous cell carcinoma, pure spindle cell carcinoma, metaplastic carcinoma with heterologous mesenchymal differentiation (e.g., chondroid, osseous, rhabdomyoid and neuroglial), fibromatosis-like metaplastic carcinoma, low-grade adenosquamous carcinoma, mixed metaplastic carcinoma [2]. Mixed metaplastic carcinoma is defined as a tumor with different metaplastic components or a mixture of metaplastic and conventional adenocarcinoma components. The cutoff value of the metaplastic component is not indicated in the WHO classification. However, considering that IDC-NST may have a tiny metaplastic component, we defined it as the metaplastic component ≥ 10%, and if the component ≥ 90% is pure MpBC.

Excepted for some low-grade subtypes, MpBCs often have large tumors and high grades and tend to have distant metastases compared to lymph node metastases. The absence of HR and HER2 expression and insensitivity to chemoradiotherapy lead to its poor prognosis. Given its rarity, most studies are limited to small samples and are known little. Many findings have shown that MpBC has shorter survival than IDC-NST [3–8], but several have remained different conclusions [9, 10]. Considered without standardized and effective treatments for MpBC, it remains a challenge to treat it. Further exploration of new treatment modalities for MpBC is necessary. We aimed to analyze the clinicopathological features, survival outcomes and treatments of MpBC.

## Materials and methods

### Population

This study collected 217 cases of MpBC patients treated at Tianjin Medical University Cancer Hospital from 2010 to 2017. In order to compare the differences between MpBC and IDC-NST, we collected IDC-NST patients from our hospital during the same period. The criteria for inclusion were (1) Female, (2) Age ≥ 18 years, (3) Pathologically confirmed MpBC/IDC-NST, (4) No history of other malignancy, (5) Non-IV stage.

### Variables

The clinicopathological characteristics used in the analysis included age at diagnosis, Nottingham grade, post-operative TNM stage (if not, use clinical stage), molecular subtype, ER status, PR status, HER2 status, Ki-67 index, P53 status, surgery type, neoadjuvant treatment, chemoradiotherapy. The judgement of TNM stage of breast cancer was followed by the American Joint Committee on Cancer (AJCC) guideline. HER2-positive implied an immunohistochemistry (IHC) score of 3 + or 2 + but with the CEP-17 ratio ≥ 2.0 using fluorescent in situ hybridization (FISH).

### Outcomes

Relapse-free Survival (RFS) was the time interval from radical treatment to tumor progression (recurrence or metastasis) or death from this disease. Breast cancer-specific survival (BCSS) was the interval between breast cancer diagnosis and death from this disease or the latest follow-up visit.

### Statistical analysis

The clinicopathological characteristics of MpBC and IDC-NST were analyzed for comparison using X^2^ and Fisher's exact tests. PSM was used to correct objective differences between the MpBC and IDC-NST groups with the caliper value of 0.01. The Kaplan–Meier curve and log-rank test were used to assess and compare clinical outcomes. Univariate and multivariate COX proportional models were used to acquire prognostic factors for MpBC. Graphpad prism (8.0) was used for plotting, PSM was performed using R software (4.2.0), and the rest were analyzed using SPSS (26.0). Every test was two-sided. When *P* value < 0 0.05, which considered it had statistical significance.

## Result

### Clinicopathological characteristics of MpBC

A total of 243 MpBC patients were collected; 15 cases of double primary cancer, two cases of stage IV at diagnosis and five cases with less than six months of follow-up were removed; In addition, two cases of adenosquamous carcinomas and four cases fibromatosis-like metaplastic carcinomas were too few to analysis. The remaining 217 patients were subsumed. As shown in Table 1, most patients (58.1%, *n* = 126) were postmenopausal women, and the median age at diagnosis was 53 years (range 25–87 years). Most tumors had AJCC stage II (63.1%, *n* = 137), T2 size (62.2%, *n* = 135) and without lymph node metastases (71.9%, *n* = 156). The preference for grade 3 (78.3%, *n* = 170) and high Ki-67 index (≥ 30%) (84.9%, *n* = 182). Of all the pathological subtypes, 45.1% (*n* = 98) were mixed subtypes, 27.2% (*n* = 59) were MpBC with mesenchymal differentiation, 15.2% (*n* = 33) and 12.4% (*n* = 27) were pure squamous and pure spindle subtypes. The majority (69.6%, *n* = 151) were HR-HER2- tumors, 24.0% (*n* = 52) were HR + HER2- tumors, 5.1% (*n* = 11) and 1.4% (*n* = 3) were HR-HER2 + and HR + HER2 + tumors, respectively. Most first preferred surgery, with only 7.4% (*n* = 16) receiving neoadjuvant treatments. Nearly all (93.5%, *n* = 203) received chemotherapy after surgery, and 28.1% (*n* = 61) received radiotherapy. Among the chemotherapy regimens, 39.2% (*n* = 85) of patients received the anthracyclines followed by taxanes (A–T) regimen, with 31.8% (*n* = 69) for the combined taxanes and anthracyclines (T+A) regimen, 11.5% (*n* = 25) for the taxanes or anthracyclines (T/A) regimen and 4.6% (*n* = 12) for the platinum-containing regimen.

**Table 1:** Clinicopathological characteristics of MpBC and IDC-NST in a 1:1 ratio matching

		MpBC (*N* = 217) (%)	IDC-NST (*N* = 217) (%)	*P* value
Age at diagnosis				0.998
< 40 years		25(11.5)	24(11.1)	
40 ~ 65 years		168(77.4)	169(77.9)	
> 65 years		24(11.1)	24(11.1)	
Family history				
Yes		57(26.3)	NA	
No		160(73.7)	NA	
Menopausal status				
Premenopausal		91 (41.9)	NA	
Postmenopausal		126(58.1)	NA	
AJCC stage				0.993
0		1(0.5)	1(0.5)	
I		48(22.1)	50(23.0)	
II		137(63.1)	134(61.8)	
III		31(14.3)	32(14.7)	
T stage				0.998
T0		1(0.5)	1(0.5)	
T1		63(29.0)	66(30.4)	
T2		135(62.2)	133(61.3)	
T3		15(6.9)	14(6.5)	
T4		3(1.4)	3(1.4)	
N stage				0.994
N0		156(71.9)	156(71.9)	
N1		37(17.1)	37(17.1)	
N2		11(5.1)	12(5.5)	
N3		13(6.0)	12(5.5)	
Nottingham grade				1.000
Grade 2		47(21.7)	48(22.1)	
Grade 3		170(78.3)	169(77.9)	
Histological type				
Pure	Pure Squamous	33(15.2)	NA	
	Pure Spindle	27(12.4)	NA	
With mesenchymal differentiation		59(27.2)	NA	
Mixed	Mixed squamous	53(24.4)	NA	
	Mixed spindle	45(20.7)	NA	
Molecular type				0.997
HR-HER2-		151(69.6)	153(70.5)	
HR + HER2-		52(24.0)	50(23.0)	
HR-HER2 +		11(5.1)	11(5.1)	
HR + HER2 +		3(1.4)	3(1.4)	
HR status				0.912
Negative		162 (74.7)	164(75.6)	
Positive		55(25.3)	53(24.4)	
HER2 status				1.000
Negative		203(93.5)	203(93.5)	
Positive		14(6.5)	14(6.5)	
KI-67 index				
< 30%		28(12.9)	NA	
≥ 30%		182(84.9)	NA	
Unknown		7(3.2)	NA	
P53 status				
Positive		120(55.3)	NA	
Negative		81(37.3)	NA	
Unknown		16(7.3)	NA	
Neoadjuvant treatment				0.701
Yes		16(7.4)	13(6.0)	
No		201 (92.6)	204(94.0)	
Surgery type				0.985
Mastectomy		193(88.9)	192(88.5)	
Breast-conserving surgery		18(8.3)	19(8.8)	
Lumpectomy		6(2.8)	6(2.8)	
Chemotherapy				1.000
Yes		203(93.5)	203(93.5)	
No		14(6.5)	14(6.5)	
Radiotherapy				1.000
Yes		61(28.1)	61(28.1)	
No		156(71.9)	156(71.9)	
Chemotherapy regimen				
T+A		69(31.8)	NA	
A−T		85(39.2)	NA	
T/A		25(11.5)	NA	
Platinum-containing		12(5.5)	NA	
Others		12(5.5)	NA	

T+A combined taxanes and anthracyclines regimens; A-T anthracyclines followed by taxanes regimens; T/A taxanes and anthracyclines regimens taxanes or anthracyclines regimens; *NA* not available

### Survival analysis of MpBC

**Fig. 1:**
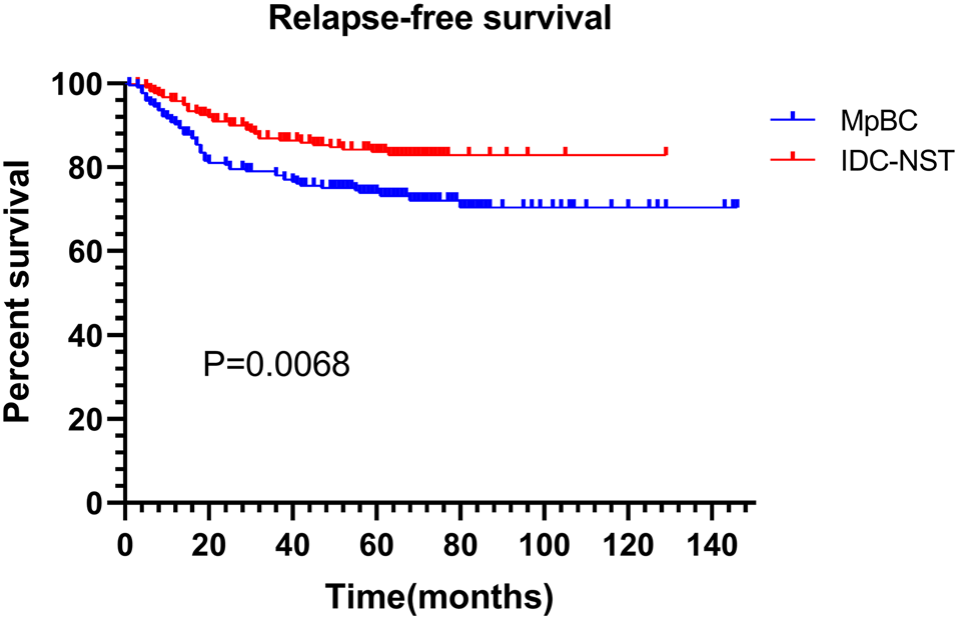
RFS between MpBC and matched IDC-NST

**Fig. 2:**
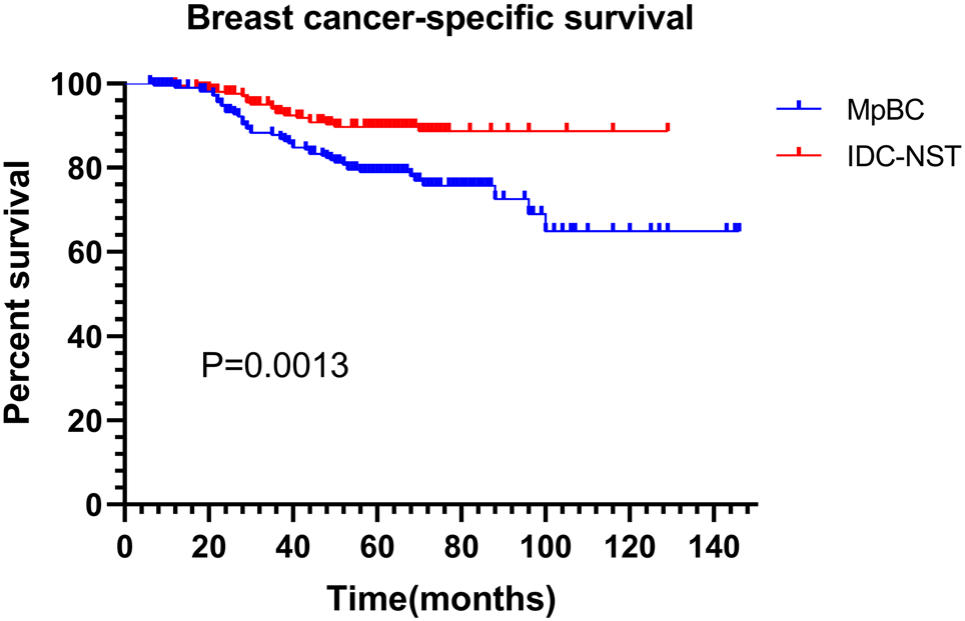
BCSS between MpBC and matched IDC-NST

The median follow-up time for patients was 61 months (range 6–146 months). The 5-year RFS and BCSS were 73.8 and 79.0%, and the 10-year RFS and BCSS were 70.4 and 64.9%, respectively (Figs. 1 and 2). 26.3% of patients (*n* = 57) had recurrences, and 22.1% (*n* = 48) had died of this disease. The most common sites of metastasis were lung (*n* = 30), bone (*n* = 19), pleural effusion or ascites (*n* = 13), chest wall (*n* = 11), liver (*n* = 7) and brain (*n* = 7). Prognostic factors for MpBC were evaluated using the Cox regression model. In univariate analysis, AJCC stage (*P* = 0.006), T size 2-5 cm (*P* = 0.010), lymph node metastases (*P* = 0.018), mixed subtype (pure vs. mixed *P* = 0.003; with mesenchymal differentiation vs. mixed *P* = 0.005) and chemotherapy (*P* = 0.003) were considered unfavorable for RFS. Age (*P* = 0.003), AJCC stage (*P* = 0.002), T size 2–5 cm (*P* = 0.005), lymph node metastases (*P* = 0.006), mixed subtype (pure vs. mixed *P* = 0.003; with mesenchymal differentiation vs. mixed *P* = 0.005) and chemotherapy (*P* = 0.002) were associated with BCSS. To eliminate confounding factors, variables with *P* value < 0.1 were included in the multivariate analysis model. Only AJCC stage (stage 0–I as reference, stage II HR = 2.698 95%CI (1.046–6.956) *P* = 0.040; stage III HR = 4.294 95%CI (1.463–12.600) *P* = 0.008), histological subtype (pure vs. mixed HR = 2.626 95%CI (1.253–5.502) *P* = 0.011; with mesenchymal differentiation vs. mixed HR = 2.136 95%CI (1.043–4.371) *P* = 0.038) and chemotherapy (HR = 0.271 95%CI (0.126–0.584) *P* = 0.001) were independent indicators for RFS. AJCC stage (stage 0–I as reference, stage II HR = 5.439 95%CI (1.286–22.999) *P* = 0.021; stage III HR = 10.080 95%CI (2.153–47.199) *P* = 0.003), histological subtype (pure vs. mixed HR = 2.731 95%CI (1.183–6.305) *P* = 0.019) and chemotherapy (HR = 0.260 95%CI (0.117–0.576) *P* = 0.001) were independent indicators for BCSS (Tables 2 and 3). Unlike common invasive breast cancer, we did not observe the significant association between HR and HER2 status and survival outcomes. In addition, radiotherapy did not improve RFS and BCSS.

**Table2:** Univariate Cox regression analysis of prognostic factors for MpBC patients

	RFS	*P* value	BCSS	*P* value
	Hazard ratio (95%CI)		Hazard ratio (95%CI)	
Age at diagnosis	1.020(0.995–1.045)	0.115	1.040(1.013–1.068)	0.003*
Family history				
No	Ref		Ref	
Yes	0.953 (0.528–1.718)	0.872	0.894(0.465–1.719)	0.738
Menopausal status				
Premenopausal	Ref		Ref	
Postmenopausal	0.868(0.515–1.465)	0.597	1.068(0.599–1.906)	0.823
AJCC stage		0.006*		0.002*
0–I	Ref		Ref	
II	3.193(1.258–8.104)	0.015*	6.603(1.586–27.495)	0.009*
III	5.428(1.931–15.252)	0.001*	12.841(2.869–57.474)	0.001*
T size		0.033*		0.015*
≤ 2 cm	Ref		Ref	
2 ~ 5 cm	2.581(1.257–5.298)	0.010*	3.462(1.462–8.196)	0.005*
> 5 cm	2.579(0.863–7.703)	0.090	3.288(0.926–11.675)	0.066
Lymph node metastases		0.018*		0.006*
N0	Ref		Ref	
N1	1.963(1.050–3.668)	0.035*	1.995(1.006–3.956)	0.048*
N2–3	2.363(1.163–4.802)	0.017*	3.055(1.469–6.354)	0.003*
Nottingham grade				
Grade 2	Ref		Ref	
Grade 3	1.007(0.533–1.904)	0.983	1.341(0.628–2.865)	0.449
Histological type		0.001*		0.001*
Pure vs. with mesenchymal differentiation	1.112(0.452–2.737)	0.817	1.111(0.402–3.065)	0.840
Pure vs. mixed	3.036(1.467–6.283)	0.003*	3.412(1.504–7.741)	0.003*
With mesenchymal differentiation vs. mixed	2.730(1.360–5.483)	0.005*	3.073(1.409–6.699)	0.005*
HR status				
Negative	Ref		Ref	
Positive	1.576(0.908–2.734)	0.106	1.287(0.690–2.399)	0.427
HER2 status				
Negative	Ref		Ref	
Positive	0.493(0.120–2.024)	0.327	0.580(0.141–2.393)	0.451
KI-67 index				
< 30%	Ref		Ref	
≥ 30%	1.030(0.467–2.274)	0.941	1.384(0.544–3.523)	0.495
P53 status				
Negative	Ref		Ref	
Positive	1.454(0.818–2.584)	0.202	1.213(0.661–2.227)	0.533
Neoadjuvant treatment				
No	Ref		Ref	
Yes	1.655(0.710–3.860)	0.243	1.680 (0.665–4.244)	0.273
Surgery type		0.354		0.171
Mastectomy	Ref		Ref	
Breast-conserving surgery	0.566 (0.177–1.816)	0.339	0.663(0.205–2.141)	0.492
Lumpectomy	1.838(0.573–5.895)	0.306	2.772(0.856–8.977)	0.089
Chemotherapy				
No	Ref		Ref	
Yes	0.327(0.155–0.691)	0.003*	0.307(0.144–0.658)	0.002*
Radiotherapy				
No	Ref		Ref	
Yes	0.854(0.474–1.541)	0.601	0.970 (0.521–1.808)	0.924

**Table 3:** Multivariate Cox regression analysis of prognostic factors for MpBC patients

	RFS	*P* value	BCSS	*P* value
	Hazard ratio (95%CI)		Hazard ratio (95%CI)	
AJCC stage		0.029*		0.011*
0-I	Ref		Ref	
II	2.698(1.046–6.956)	0.040*	5.439 (1.286–22.999)	0.021*
III	4.294(1.463–12.600)	0.008*	10.080(2.153–47.199)	0.003*
Histological type		0.012*		0.027*
Pure vs. with mesenchymal differentiation	1.229(0.498–3.037)	0.654	1.321(0.476–3.666)	0.593
Pure vs. mixed	2.626(1.253–5.502)	0.011*	2.731(1.183–6.305)	0.019*
With mesenchymal differentiation vs. mixed	2.136(1.043–4.371)	0.038*	2.067(0.931–4.591)	0.075
Chemotherapy				
No	Ref		Ref	
Yes	0.271(0.126–0.584)	0.001*	0.260(0.117–0.576)	0.001*

### Analysis of the prognosis differences between MpBC and IDC-NST

Since baseline imbalance may induce outcome discrepancies, we used the PSM to 1:1 matching, 217 IDC-NST patients were screened in comparison with MpBC patients, and the chosen factors were matched well between the two groups (Table 1**)**. It can be seen that MpBC had inferior survival compared with IDC-NST, with 5-year RFS of 73.8 and 83.6% (HR = 1.177 95%CI (1.171–2.676) *P* = 0.0068), and 5-year BCSS of 79.0% and 89.7% (HR = 2.187 95%CI (1.357–3.523) *P* = 0.0013). (Figs. 1 and 2).

### Subgroup analysis of MpBC

**Fig. 3:**
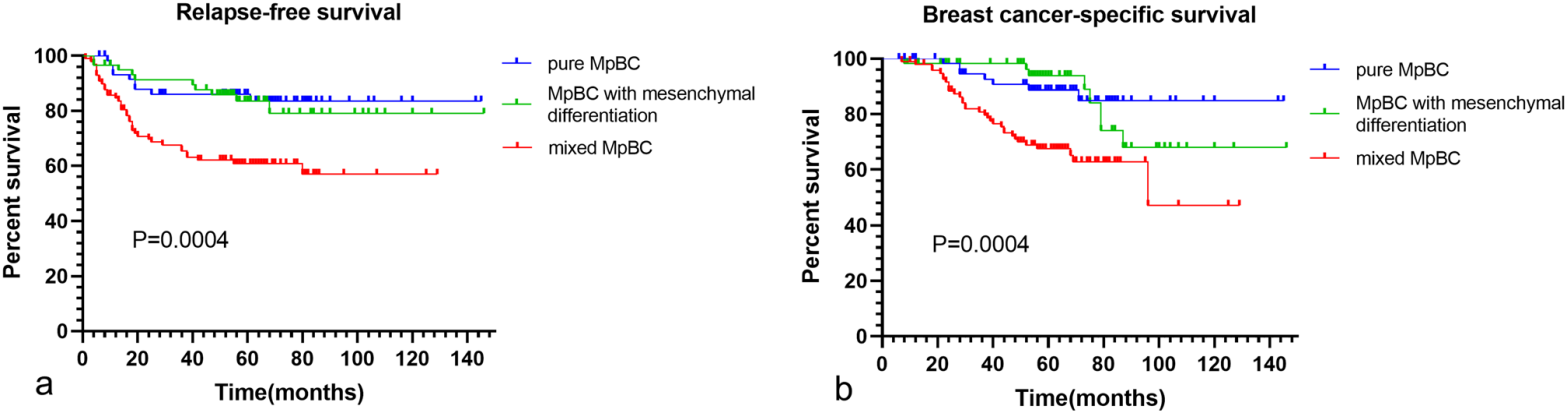
RFS and BCSS between histological subtypes of MpBC

We drew their survival curves according to the histological subtypes (Fig. 3). Mixed MpBC had a poorer prognosis than MpBC with mesenchymal differentiation and pure MpBC, with 5-years RFS of 60.7, 83.5 and 86.0% (*P* = 0.0004) and 5-years BCSS of 67.5, 93.9 and 88.7%, respectively (*P* = 0.0004). The survival outcomes of with mesenchymal differentiation and pure subtypes had no statistical difference. The clinicopathological characteristics of different subtypes were compared using the *X*^2^ and Fisher's exact test (Table 4). The mixed subtype preferred higher AJCC stages (*P* = 0.001), bigger tumors (*P* = 0.005), more lymph node involvements (*P* = 0.024), higher Nottingham grade (*P* = 0.033), higher Ki-67 index (*P* < 0.001) and more HER2 expression (*P* = 0.032).

**Table 4:** Clinicopathological characteristics of MpBC between histological subtypes

	Pure (*N* = 60) (%)	With mesenchymal differentiation (*N* = 59) (%)	Mixed (*N* = 98) (%)	*P* value
Age at diagnosis				0.545
< 40 years	5(8.3)	9(15.3)	11(11.2)	
40 ~ 65 years	46(76.7)	46(78.0)	76(77.6)	
> 65 years	9(15.0)	4(6.8)	11(11.2)	
Family history				0.278
No	45(75.0)	39(66.1)	76(77.6)	
Yes	15(25.0)	20(33.9)	22(22.4)	
Menopausal status				0.602
Premenopausal	24(40.0)	28(47.5)	39(39.8)	
Postmenopausal	36(60.0)	31(52.5)	59(60.2)	
AJCC stage				0.001*
0–I	17(28.3)	21(35.6)	11(11.2)	
II	39(65.0)	33(55.9)	65(66.3)	
III	4(6.7)	5(8.5)	22(22.4)	
T size				0.005*
≤ 2 cm	21(35.0)	26(44.1)	17(17.3)	
2 ~ 5 cm	36(60.0)	28(47.5)	71(72.4)	
> 5 cm	3(5.0)	5(8.5)	10(10.2)	
Lymph node metastases				0.024*
N0	50(83.3)	46(78.0)	60(61.2)	
N1	7(11.7)	9(15.3)	21(20.4)	
N2–3	3(5.0)	4(6.8)	17(17.3)	
Nottingham grade				0.033*
Grade 2	19(31.7)	14(23.7)	14(14.3)	
Grade 3	41(68.3)	45(76.3)	84(85.7)	
HR status				0.266
Negative	47(78.3)	47(80.0)	68(69.4)	
Positive	13(21.7)	12(20.0)	30(30.6)	
HER2 status				0.032*
Negative	58(96.7)	58(98.3)	87(88.8)	
Positive	2(3.3)	1(1.7)	11(11.2)	
KI-67 index				P < 0.001*
< 30%	17(28.3)	5(8.5)	6(6.1)	
≥ 30%	41(68.3)	50(84.7)	91(92.9)	
Unknown	2(3.3)	4(6.8)	1(1.0)	
P53 status				0.565
Negative	23(38.3)	24(40.7)	34(34.7)	
Positive	32(53.3)	29(49.2)	59(60.2)	
Unknown	5(8.3)	6(10.2)	5(5.1)	
Neoadjuvant treatment				0.942
No	55(91.7)	55(93.2)	91(92.9)	
Yes	5(8.3)	4(6.8)	7(7.1)	
Surgery type				0.634
Mastectomy	53(88.3)	51(86.4)	89(90.8)	
Breast-conserving surgery	4(6.7)	7(11.9)	7(7.1)	
Lumpectomy	3(5.0)	1(1.7)	2(2.0)	
Chemotherapy				1.000
No	4(6.7)	4(6.8)	6(6.1)	
Yes	56(93.3)	55(93.2)	92(93.9)	
Radiotherapy				0.621
No	46(76.7)	41(69.5)	69(70.4)	
Yes	14(23.3)	18(30.5)	29 (29.6)	

**Fig. 4:**
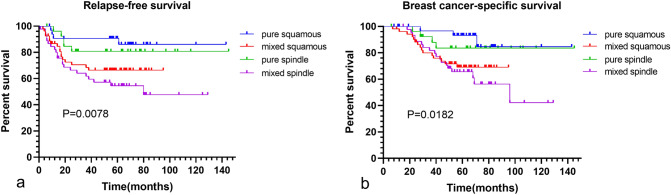
RFS and BCSS between pure and mixed subtypes of MpBC

Further dividing mixed MpBC, 53 cases were mixed squamous subtypes and 45 cases were mixed spindle subtypes. For the mixture component, 96 cases were IDC-NST, one case was invasive carcinoma with neuroendocrine differentiation and one case was invasive carcinoma with apocrine differentiation. 95 cases had only one metaplastic component, two cases had squamous and spindle elements, and one case had spindle and osseous elements. We performed subgroup analysis for pure and mixed MpBC**.** Either squamous or spindle MpBC, mixed forms had worse outcomes than pure forms (Fig. 4). For mixed and pure squamous MpBC, their 5-year RFS were 66.4% and 90.6% (HR = 2.539 95%CI (1.061–6.075) *P* = 0.0363) and 5-year BCSS were 69.1 and 93.1% (HR = 2.805 95%CI (1.096–7.180) *P* = 0.0315). For mixed and pure spindle MpBC, their 5-year RFS were 54.5 and 80.8% (HR = 2.492 95%CI (1.136–5.467) *P* = 0.0227) and 5-year BCSS were 65.7 and 83.5% (HR = 2.449 95%CI (1.034–5.801) *P* = 0.0418). In contrast, the prognoses of pure squamous and pure spindle subtypes showed no significant differences. The clinicopathological characteristics between pure and mixed subtypes are shown in Table 5. Mixed squamous MpBC had higher AJCC stages (*P* = 0.034), larger tumors (*P* = 0.027), higher Nottingham grade (*P* = 0.026), and higher Ki-67 index (*P* = 0.009) than the pure subtype. Compared to the pure spindle subtype, mixed had higher AJCC stages (*P* = 0.036), more lymph node metastases (*P* = 0.019), more HR expression (*P* = 0.017) and higher Ki-67 index (*P* = 0.020).

**Table 5:** Clinicopathological characteristics of MpBC between pure and mixed subtypes

	Pure squamous (*N* = 33) (%)	Mixed squamous (*N* = 53) (%)	*P* value	Pure spindle (*N* = 27) (%)	Mixed Spindle (*N* = 45) (%)	*P* value
Age at diagnosis			0.639			0.455
< 40 years	4(12.1)	5(9.4)		1(3.7)	6(13.3)	
40 ~ 65 years	24(72.7)	43(81.1)		22(81.5)	33(73.3)	
> 65 years	5(15.2)	5(9.4)		4(14.8)	6(13.3)	
Family history			0.864			0.720
No	25(75.8)	41(77.4)		20(74.1)	35(77.8)	
Yes	8(24.2)	12(22.6)		7(25.9)	10(22.2)	
Menopausal status			0.376			0.352
Premenopausal	15(45.5)	19(35.8)		9(33.3)	20(44.4)	
Postmenopausal	18(54.5)	34(64.3)		18(66.7)	25(55.6)	
AJCC stage			0.034*			0.036*
0–I	11(33.3)	6(11.3)		6(22.2)	5(11.1)	
II	19(57.6)	37(69.8)		20(74.1)	28(62.2)	
III	3(9.1)	10(18.9)		1(3.7)	12(26.7)	
T size			0.027*			0.622
≤ 2 cm	15(45.4)	10(18.9)		6(22.2)	7(15.6)	
2 ~ 5 cm	17(51.5)	39(73.6)		19(70.4)	32(71.1)	
> 5 cm	1(3.0)	4(7.5)		2(7.4)	6(13.3)	
Lymph node metastases			0.108			0.019*
No	26(78.8)	33(62.3)		24(88.9)	27(60.0)	
Yes	7(21.2)	20(37.7)		3(11.1)	18(20.0)	
Nottingham grade			0.026*			0.155
Grade 2	11(33.3)	7(13.2)		8(29.6)	7(15.6)	
Grade 3	22(66.7)	46(86.8)		19(70.4)	38(84.4)	
HR status			0.492			0.017*
Negative	22(66.7)	39(73.6)		25(92.6)	29(64.4)	
Positive	11(33.3)	14(26.4)		2(7.4)	16(35.6)	
HER2 status			0.664			0.150
Negative	31(93.9)	47(88.7)		27(100)	40(88.9)	
Positive	2(6.1)	6(11.3)		0(0)	5(11.1)	
KI-67 index			0.009*			0.020*
< 30%	8(24.2)	2(3.8)		9(33.3)	4(8.9)	
≥ 30%	24(72.7)	51(96.2)		17(63.0)	40(88.9)	
Unknown	1(3.0)	0(0)		1(3.7)	1(2.2)	
P53 status			0.743			0.543
Negative	10(30.3)	15(28.3)		13(48.1)	19(42.2)	
Positive	21(63.6)	37(69.8)		13(48.1)	22(48.9)	
Unknown	3(9.1)	1(1.9)		1(3.7)	4(8.8)	
Neoadjuvant treatment			0.509			0.720
No	29(87.9)	50(94.3)		26(96.3)	41(91.1)	
Yes	4(12.1)	3(5.7)		1(3.7)	4(8.9)	
Surgery type			0.180			0.149
Mastectomy	32(97.0)	47(88.7)		21(77.8)	42(93.3)	
Breast-conserving surgery	0(0)	5(9.4)		4(14.8)	2(4.4)	
Lumpectomy	1(3.0)	1(1.9)		2(7.4)	1(2.2)	
Chemotherapy			1.000			1.000
No	2(6.1)	2(3.8)		2(7.4)	4(8.8)	
Yes	31(93.9)	51(96.2)		25(92.6)	41(91.2)	
Radiotherapy			0.627			0.086
No	24(72.7)	41(77.4)		22(81.5)	28(62.2)	
Yes	9(27.3)	12(22.6)		5(18.5)	17(37.8)	

### Treatment strategies of MpBC

MpBC responded poorly to neoadjuvant chemotherapy (NAC). Of all 16 patients who received NAC, only 6.3% (*n* = 1) patients achieved pathological complete response (pCR) and 56.3% (*n* = 9) changed treatment regimens due to NC (no significant change) or PD (progressive disease), ultimately 37.5% (*n* = 6) occurred recurrences or metastases.

93.6% of patients (*n* = 15) preferred the T+A regimen, and all nine patients had the platinum-containing regimen as the second-line regimen.

**Fig. 5:**
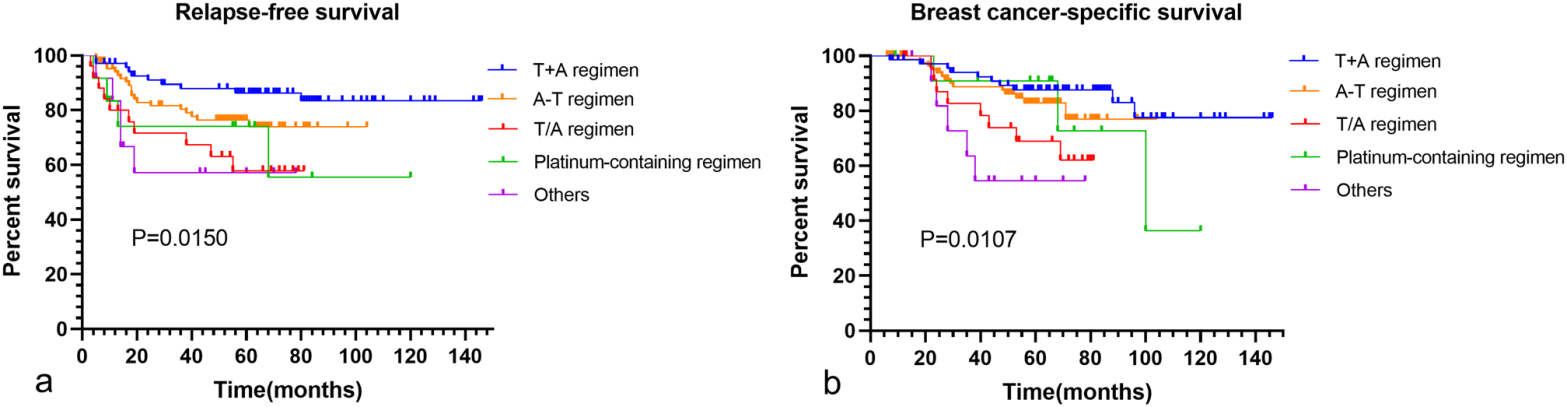
RFS and BCSS between different chemotherapy regimens

For the subgroup analysis of adjuvant therapy, receiving the T+A regimen had more prolonged RFS and BCSS than receiving the T/A regimen (*P* = 0.003 and *P* = 0.014). Receiving other regimens (including capecitabine; mesna, doxorubicin, ifosfamide and dacarbazine (MAID) and unknown regimens) had shorter RFS and BCSS compared to receiving the T+A regimen (*P* = 0.004 and *P* = 0.002) (Fig. 5). In contrast, receiving A−T or platinum-containing regimens had inferior RFS compared to receiving the T+A regimen but without statistical significance (*P* = 0.093 and *P* = 0.052). We did not observe survival differences in BCSS between receiving A–T or platinum-containing regimens and the T+A regimen (*P* = 0.348 and *P* = 0.297).

**Fig. 6:**
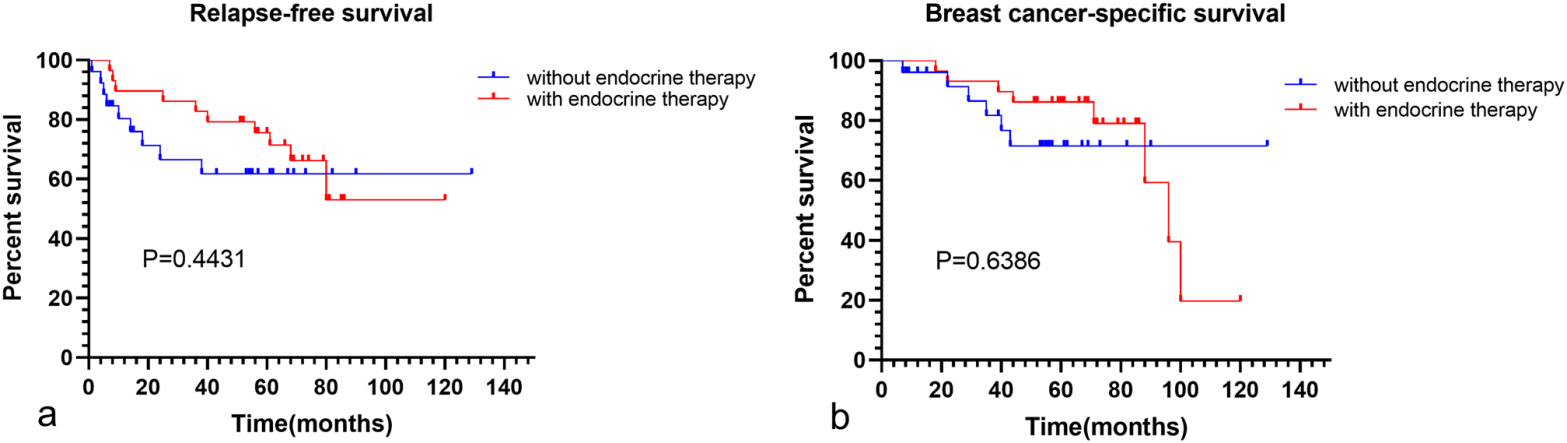
RFS and BCSS between endocrine therapies

Endocrine and targeted therapy may be required for HR-positive and HER2-positive patients. Of HR-positive patients, more than half (*n* = 29) used endocrine therapy (using more than 6 months). The K–M curves of the two groups with or without endocrine therapy were shown no statistical differences between these two cohorts** (**Fig. 6). Of HER2-positive patients, 57.1% (*n* = 8) were treated with anti-HER2 therapy (using more than twice), and only two without targeted therapy had recurrence and death.

## Discussion

MpBC is a heterogeneous subtype of breast cancer that they are highly invasive and more prone to distant metastases except for fibromatosis-like metaplastic carcinoma and low-grade adenosquamous carcinoma [11].

There are controversies about prognoses of other histological subtypes. MpBCs are classified as monophasic (only one metaplastic component) or biphasic (both metaplastic and non-metaplastic components) tumors. The former includes pure SpCC and SqCC, and the latter includes mixed MpBC and MpBC with heterologous mesenchymal differentiation. In monophasic MpBC, Rakha et al. [9] and McCart et al. [12] also indicated that squamous cell carcinoma had more prolonged survival while Tadros et al. [8] reported a worse outcome for it. In biphasic MpBC, we found that patients with mesenchymal differentiation had better RFS and BCSS. Tadros et al. [8], Rakha et al. [9] and Takala et al. [13] also demonstrated this finding. The lack of PI-3 kinase and Ras-Map kinase aberrations and TERT promoter mutations may account for their less aggressive feature [14]. For mixed MpBC, previously, scholars generally agreed that it contains two or more metaplastic components. It revealed that conventional mixed MpBC had a worse outcome [13, 15, 16], and the prognosis was worse with the number of components increasing [12]. After publishing the 5th edition of the WHO classification, metaplastic carcinoma with adenocarcinoma component was also classified as a mixed subtype. In this study, most mixed MpBCs were the mixture of one metaplastic component with invasive ductal carcinoma. The mixed subtypes with either squamous or spindle differentiation had shorter survival outcomes than the pure subtypes. Consistent with our findings, recent research of 39 squamous cell carcinomas showed that invasive carcinoma with squamous differentiation had a worse prognosis and was more aggressive than pure squamous subtype [17]. A similar phenomenon was seen in uterine carcinosarcoma, where Renske et al. found that most metastases (72%) and vascular invasion (70%) were caused by epithelial components rather than mesenchymal components [18]. These phenomena suggest the need for further research into the origin of MpBC. The conversion theory is generally accepted, which asserts that the mesenchymal component is derived from the epithelial component through metaplastic progress. Some studies confirmed that when MpBC with the invasive ductal component, the genetic progression and evolution of its metaplastic component was from its paired invasive ductal carcinoma [14, 19]. The poorer prognosis of mixed MpBC with invasive carcinoma may imply that the epithelial component drives the high proliferation of MpBC. For example, vascular endothelial growth factor [20] and matrix metalloproteinase-7 (MMP-7) [21] were higher expressed in the epithelial component compared to the mesenchymal component, all of which contribute to its invasion and metastasis. In contrast, a study of mixed histological subtypes of invasive breast carcinoma showed no prognostic difference between pure and mixed MpBC [22], possibly due to the small sample sizes and subtype discrepancies included in their cohort. The different conclusions may reflect the different classification criteria and sample sizes of each study, furthermore, it is recommended that pathologists should record the morphology and proportion of each component at diagnosis in order to make subsequent therapeutic options.

The prognostic significance of clinicopathological features has been reported a lot, but there is no consensus on the most useful. Multiple reports, including our study, have found the mixed subtype to be an unfavorable factor [12, 13, 15, 16]. Many authors regarded tumor size was correlated with survival [12, 13, 15, 23, 24]. However, one multi-center research indicated that only lymph node status and lymphovascular invasion influenced prognosis, not tumor size and histological grade [9]. A series of studies with the SEER database proved that age and TNM stage were all independent prognostic factors [25–28]. Chemotherapy was effective for survival, but there remains controversy in radiotherapy. Multiple research confirmed that radiotherapy could improve OS or BCSS[6, 29, 30], but some considered it of limited benefit. For example, Leyrer et al. showed it decreased the local–regional but not distant recurrence [31]. Haque et al. suggested that mastectomy with radiotherapy was associated with improved OS only in the high-risk (T3–4 or node-positive) but lumpectomy with it both in high or low-risk (T1–2N0) cohorts[32]. Similar to us, a multi-center study showed no association between radiotherapy and outcomes[9]. These biases may be caused by objective differences between Asian and Western cohorts, with only 5.6–28.1% receiving radiotherapy in our and other Asian studies[3] but 37.9–92.9% in Western studies[33]. Besides, it showed that surgery type [34, 35] and race [5, 25] might affect clinical outcomes.

MpBCs are often triple-negative breast cancers, but up to 23 and 5.2% acquired HR and HER2 according to an analysis for SEER [36], which was broadly similar to our results (25.3 and 6.5%). HR-positive breast cancer is usually considered to have a better outcome. However, recent findings, including ours, found that HR-positive status and endocrine therapy might not provide a survival benefit for MpBC patients [25, 27]. Wu et al. [37] observed that HR-positive increased the death risk compared to HR-negative subtypes. In contrast, one research based on NCDB held the opposite view suggesting that endocrine therapy can improve OS [7]. In addition, our study showed HER2 status was not associated with prognosis. Interestingly, one revealed that HER2-positive MpBC might derive more benefits for survival [36], but Lei et al. [38] reported that HER2-positive squamous carcinoma was associated with an inferior prognosis. Rare reports mentioned targeted therapy for MpBC. In our research, none of the eight patients receiving targeted therapy had recurred, while two of the remaining six patients occurred recurrence and death. Anti-HER2 therapy appears to improve the prognoses of HER2-positive patients. But it is not convincing due to the limited sample size. It requires us to expand the sample to prove this conclusion.

MpBC has little response to NAC, with previous studies showing the pCR rate in the range of 0–17% [7, 15, 16, 23, 24, 39]. A recent prospective trial (NCT02276443) showed the pCR rate reaching 23% [40]. In this study, only 6.3% of MpBC patients achieved pCR, which contrasts with high pCR rates in TNBC, 30–40% with T+A regimens and over 50% with platinum-containing regimens [41, 42]. It has been certified that the enrichment of the EMT pathway and the widespread expression of tumor stem cells in MpBC make them resistant to anthracycline and paclitaxel drugs [43, 44]. Besides, the up-regulation of FOXC1 plays an important part in this process [45]. For the adjuvant treatment, the T+A regimen substantially improved the RFS and BCSS of MpBC. Unlike it is reported to be effective for TNBC, the platinum-containing regimen did not have a better outcome than the T+A regimen in our analysis, possibly due to less use and clinician selection bias, with a preference for platinum when large tumors or lymph node metastases. However, it seems to be improved when using the dense dose. A retrospective review reported that weekly paclitaxel and platinum chemotherapy could prolong survival significantly [46].

Because of deriving less benefit from chemoradiotherapy and endocrine therapy, exploring efficient treatments becomes an urgent need. With more researchers finding the prevalence of PI3K mutations in MpBC [12, 47–49], inhibitors of the PI3K/AKT/mTOR pathway combined with chemotherapy or targeted therapy have provided effective therapeutic methods [50, 51]. Furthermore, MpBC has been reported that frequent expression of programmed death-ligand 1 (PD-L1) which is similar to TN-IDC [44, 47, 48, 52, 53]. Several clinical trials (NCT02834013, NCT02752685) using anti-PD-1/PD-L1 antibodies in advanced MpBC patients have achieved explicit survival benefits [54, 55]. Other studies have proved that TERT promoter hotspot mutations [56] and the RPL39 A14V mutations [57] occur at high frequency in MpBC, and MYC amplification is highly enriched after metastasis [58]. These may become potential targets for treating MpBC in the future.

This study presented the clinicopathological characteristics, treatment strategies and prognoses of 217 patients with MpBC from our institution over eight years and compared them with IDC-NST. This study has some limitations: Firstly, it may have some selection bias because of its retrospective analysis and limited sample size. Secondly, this research is single-center, and the cases included may have local characteristics and limitations. Finally, some indicators such as Ki-67, P53 and menopausal status can't be matched, so there may still be an imbalance between the two cohorts. However, our study is one of the largest single institutions by far to include relatively comprehensive clinicopathological features of this rare tumor, and it’s representative and credible due to the long follow-up period and wide sample variety.

## Conclusion

MpBC is associated with shorter survival than IDC-NST. Different histological subtypes have unique clinicopathological features and survival outcomes. The mixed subtype is more aggressive than pure and with heterologous mesenchymal differentiation subtypes. It provides direction for making therapeutic guidelines in clinical. For the rare MpBC expressed HR and HER2, it seems that anti-HER2 therapy but not endocrine therapy provides benefits in survival. However, it necessary to expand samples to explore this research further.

## Data Availability

The datasets in this study can be available from the corresponding author upon reasonable request.
